# THE HEALING POWER OF COMPASSIONATE LOVE: LATER-LIFE CAREGIVING STATUS AND MENTAL HEALTH IN THE UNITED STATES

**DOI:** 10.1093/geroni/igae098.3385

**Published:** 2024-12-31

**Authors:** Nirmala Lekhak, Tirth Bhatta, Foster Kamanga, Cecilia Fernandes, Eva Kahana

**Affiliations:** University of Nevada Las Vegas, Las Vegas, Nevada, United States; University of Nevada Las Vegas, Las Vegas, Nevada, United States; University of Nevada Las Vegas, Las Vegas, Nevada, United States; PrismaHealth Children’sHospital, Richland, South Carolina, United States; Case Western Reserve University, Cleveland, Ohio, United States

## Abstract

The detrimental effects of family caregiving on mental health have been extensively established, with research focusing on emotional support as a mechanism to explain these relationships. However, previous research has not investigated such mechanisms across caregiving statuses especially among older caregivers. Furthermore, the significance of compassionate love in understanding the negative impacts of caregiving on mental health lacks attention. Using nationwide web-based data (n=1,861), we examined the unique role of compassionate love in explaining the differences in depressive symptoms, anxiety, and loneliness across caregiving status. Our ordinary least squares regression (OLS) estimates indicate that not caregivers, former caregivers, and caregivers who were not living with care-recipients (caregivers not in-residence) had significantly lower levels of anxiety, loneliness, and depressive symptoms than caregivers who were living with care-recipients (caregivers in-residence), with the most notable difference observed in loneliness. After controlling for socioeconomic status, health, and emotional support in our models, compassionate love entirely accounted for the remaining differences in depressive symptoms and anxiety between caregivers in-residence and not caregivers. Additionally, it fully explained the remaining differences in loneliness between former caregivers, caregivers not in-residence, and those living with them. Although considerably weaker (changed from b=-0.48, p< 0.001 to b=-0.38, p< 0.01), the loneliness gap between not caregivers and caregivers in-residence remained statistically significant even after accounting for love. Our findings highlight the importance of recognizing differences in caregiving status when designing future interventions to improve family caregivers’ feelings of love and mental health in later life.

